# Stigma and Its Association With Glycemic Control and Hypoglycemia in Adolescents and Young Adults With Type 1 Diabetes: Cross-Sectional Study

**DOI:** 10.2196/jmir.9432

**Published:** 2018-04-20

**Authors:** Anne-Sophie Brazeau, Meranda Nakhla, Michael Wright, Mélanie Henderson, Constadina Panagiotopoulos, Daniele Pacaud, Patricia Kearns, Elham Rahme, Deborah Da Costa, Kaberi Dasgupta

**Affiliations:** ^1^ School of Human Nutrition McGill University Montreal, QC Canada; ^2^ Department of Pediatrics McGill University Montreal, QC Canada; ^3^ Department of Exercise Science Concordia University Montréal, QC Canada; ^4^ Centre Hospitalier Universitaire Sainte-Justine Department of Pediatrics Université de Montréal Montréal, QC Canada; ^5^ BC Children’s Hospital University of British Columbia Vancouver, BC Canada; ^6^ Alberta Children’s Hospital University of Calgary Calgary, QC Canada; ^7^ Breast Cancer Action Quebec Montréal, QC Canada; ^8^ Centre for Outcomes Research and Evaluation Research Institute of the McGill University Health Centre Department of Medicine, McGill University Montreal, QC Canada

**Keywords:** type 1 diabetes, youth, young adult, social stigma, perception, glycated hemoglobin A1c

## Abstract

**Background:**

Qualitative studies in type 1 diabetes indicate that visibility of diabetes supplies, self-care, and hypoglycemia symptoms are associated with stigma and suboptimal management. This may be particularly salient in youth who face concurrent challenges such as establishing autonomy and making vocational choices.

**Objective:**

The aim of the study was to estimate stigma prevalence in youth (aged 14-24 years) with type 1 diabetes and its associations with glycemic control.

**Methods:**

Participants, recruited largely through social media, were asked to complete a Web-based survey and to send via mail capillary blood samples for glycated hemoglobin (HbA_1c_) measurement. The primary definition of stigma required endorsement of one or more of 3 stigma-specific items of the Barriers to Diabetes Adherence questionnaire. These addressed avoidance of diabetes management with friends present, difficulty telling others about diabetes diagnosis, and embarrassment in performing diabetes care with others present. Poor glycemic control was defined as HbA_1c_>9% (ie, >75 mmol/mol; measured value when available, else self-report) and/or ≥1 severe hypoglycemic episode in the previous year (reported requiring assistance from someone else during the episode). Stigma prevalence was computed (95% CI), and associations with glycemic control were evaluated (multivariate logistic regression models).

**Results:**

Among the 380 respondents, stigma prevalence was 65.5% (95% CI 60.7-70.3). Stigma was associated with a 2-fold higher odds of poor glycemic control overall (odds ratio [OR] 2.25, 95% CI 1.33-3.80; adjusted for age, sex, and type of treatment). There were specific associations with both HbA_1c_>9% (75 mmol/mol; OR 3.05, 95% CI 1.36-6.86) and severe hypoglycemia in the previous year (OR 1.86, 95% CI 1.05-3.31).

**Conclusions:**

There is a high prevalence of stigma in youth with type 1 diabetes that is associated with both elevated HbA_1c_ levels and severe hypoglycemia. Targeted strategies to address stigma are needed.

**Trial Registration:**

ClinicalTrials.gov NCT02796248; http://clinicaltrials.gov/ct2/show/NCT02796248 (Archived by WebCite at http://www.webcitation.org/6yisxeV0B)

## Introduction

Stigma related to chronic disease is a negative social judgment that leads to unwarranted rejection or exclusion. It is related to visible features of the disease or its management [1,2]. In conceptualizing stigma, it is important to consider these features, the sources of stigma (eg, individuals, groups, media), and the psychological mechanisms driving stigma such as fear, blame, or disgust [3]. Stigma is characterized by labeling, negative stereotyping, *us versus them* attitudes, and loss of status or discrimination [1]. It may be experienced or perceived, which, in turn, may engender self-stigmatization, an internalization, and acceptance of stigma. The harm that results may be psychological, social, behavioral, and medical. Chronic disease–related stigma has been studied in the context of mental illness, HIV/AIDS, and type 2 diabetes [4-6]. It has been less extensively studied in type 1 diabetes.

Type 1 diabetes is a chronic autoimmune disease with usual onset in childhood and youth. It is characterized by complex and noticeable self-management imperatives, including insulin injection or pump use, capillary blood glucose testing, and attention to meal timing, food choices, and physical activity levels. It also has strong potential for symptomatic hypoglycemia with confusion, distress, or loss of consciousness. The visibility of the equipment, blood testing, making adjustments to therapy, and hypoglycemic symptoms, if they occur, are the disease features that have the potential to lead to stigma [3,7,8]. Most studies examining stigma in type 1 diabetes have been qualitative evaluations that provide insight into the sources (eg, coworkers, family members, media) and characteristics (eg, name calling, rejection) of stigma [3,8,9]. These highlight the blame and discrimination experienced by individuals with type 1 diabetes, which may lead them to hide their condition.

Stigma may be particularly salient when combined with the challenges of adolescence and young adulthood (ie, youth). Many youth with diabetes struggle with self-esteem, body image, social role definition, and peer-related issues [10]. During adolescence, peer relationships and acceptance by friends are essential [11]. In an effort to avoid being seen as different by their peers, qualitative studies suggest that youth with type 1 diabetes may engage in passive coping strategies, such as avoidance of activities and nonadherence to treatment regimens [12-14]. These behaviors are not limited to adolescence but continue into early adulthood, a stage in life termed emerging adulthood, characterized by the challenges of establishing autonomy, personal identity, and making vocational and educational choices [15]. Emerging adults with diabetes must contend with complex developmental tasks while also dealing with their condition and its treatment [16]. There is a paucity of evidence addressing the prevalence of stigma and quantifying its consequences in youth with type 1 diabetes.

Two publications have examined stigma prevalence, one in a mixed population of people with type 1 and type 2 diabetes, in which 70% reported having experienced stigma [17] and a second that included both type 1 and type 2 diabetes patients but reported on each separately [18]. Youth were included in this latter study, but parents rather than patients completed questionnaires; 83% of parents believed that diabetes comes with social stigma. Although qualitative studies suggest that stigma is an important issue for people with type 1 diabetes, there have been no previous large-scale studies estimating stigma prevalence in youth through direct query, nor have associations with glycemic control been evaluated. To address these knowledge gaps, we conducted a cross-Canada study that incorporated social media–based recruitment, online questionnaires, and mailed-in capillary blood samples in youth (adolescents and emerging adults) with type 1 diabetes.

## Methods

### Overview

The study design and methods, described previously (Clinicaltrials.gov NCT02796248) [19], are briefly reviewed here. Procedures were approved by the Institutional Review Boards of the McGill University Health Centre, the Research Centre of the Centre Hospitalier Universitaire Sainte-Justine, the University of British Columbia, and the University of Calgary. Recruitment and data collection occurred between May 4, 2016, and January 4, 2017.

### Questionnaire

The questionnaire incorporated existing instruments and new questions formulated by our team of researchers, patient representatives, and physicians. In a pilot study, high reliability was observed with intraclass coefficients >.95 for each scale [19]. We queried demographic and clinical information (age at diagnosis, insulin pump vs multiple daily injection, hypoglycemia frequency and severity, most recent glycated hemoglobin, HbA_1c_ value), incorporated the Barriers to Diabetes Adherence in Adolescence questionnaire (BDA; 21 items; maximum score of 5) [20], and included 12 closed-ended questions we developed (informed by Browne et al’s diabetes-related stigma framework [3]), and open-ended questions (free text responses).

### Stigma Definition

Stigma was assessed using the BDA stigma subscale, the only scale available to measure stigma in adolescents with type 1 diabetes. No specific cutoffs for this subscale have been established or validated. Our aim was to determine prevalence rather than severity. Therefore, we defined stigma as an affirmative response to at least one of 3 key items on the BDA stigma subscale (score ≥2 on a 5-point Likert-type scale; alternate thresholds were also examined, see Multimedia Appendix 1). These (*I try not to deal with my diabetes in front of friends; I have a hard time telling people I have diabetes; I feel embarrassed taking care of my diabetes in front of other people*) were selected a priori by our team. These 3 items assess consequences of stigma.

Several other alternate definitions of stigma were evaluated such as providing a personal experience of feeling judged for having diabetes and combining a personal experience with endorsement of at least one of the 3 key BDA stigma subscale items.

### Self-Efficacy and Well-Being

The *Self-Efficacy for Diabetes Self-Management* measure (SEDM; 10 items; maximum score of 10; higher score indicates greater self-efficacy) [21] and the *World Health Organization-5 Well-Being index* (WHO-5 Well-Being index; 5 items; maximum score of 100; higher score indicates greater sense of well-being) [22,23] were both incorporated in our questionnaire. These tools have been validated in adolescents [21,23].

### Poor Glycemic Control

Poor glycemic control was defined as an HbA_1c_ level above 9% (75 mmol/mol) and/or at least 1 severe hypoglycemic episode in the last year, defined as requiring assistance from someone else during the episode. When only one type of HbA_1c_ measure was available (ie, self-reported vs direct measurement), this measure was used to classify into an HbA_1c_ category (ie, ≤9% vs >9%). When both types were available, the direct measurement was used. To assess agreement between types, a Pearson correlation was computed, and a Bland-Altman plot was generated.

### Recruitment

Adolescents (aged 14-18 years) and emerging adults (aged 19-24 years) with type 1 diabetes were eligible for this study (ie, youth). A comprehensive prevalence survey would have captured all youth with type 1 diabetes across Canada or used these individuals as a sampling framework and subsampled among them. However, there is no diabetes registry that reliably identifies this group of individuals in Canada. We therefore opted to partner with diabetes-related organizations to reach out to this target population through social media. Diabetes Canada and several smaller diabetes and patient organizations (see Multimedia Appendix 1) partnered with us, tweeting members about the study and publicizing it on Facebook. Some patients were approached by their medical team (in person or by email) and provided with the study website address. The study focus was described as “living with type 1 diabetes.” A purposive sample of respondents was recruited.

### Data Collection

Participants registered on the secure study website and were then emailed a link to an online consent form and questionnaire. After survey completion, they were asked if they would agree to provide a blood sample for HbA_1c_ assessment. Those who consented were mailed a kit. They received a Can $10 gift card after survey completion and a second after mailing in a capillary blood sample. HbA_1c_ was measured on these samples (DTIL Laboratories, Inc, Thomasville, GA, USA).

Survey completion time was reviewed. Our pilot study in 30 participants from patients with whom our team had direct contact indicated a mean questionnaire completion time of 20:19 min (SD 8:52), ranging from 9:13 to 39:41 [8]; therefore, if participants completed the survey in <9 min and/or had little variation in answers (eg, selecting all 1 on a 1-10 Likert scale), we emailed a request to call us directly through a 1-800 number so that responses could be verified. In the absence of such verification, respondents were excluded.

### Data Analysis

Means and SDs or proportions were used to report participant characteristics, as appropriate. Stigma prevalence was calculated with 95% CI overall, by sex, and separately among adolescents (ie, aged 14-18 years) and emerging adults (ie, aged 19-24 years). Logistic regression models were constructed to examine associations between stigma and poor glycemic control (primary outcome). Variables considered for inclusion were gender, age, diabetes duration, and insulin administration method. In an alternate model, SEDM and well-being were also included. Linear regression models were constructed to examine associations of stigma with SEDM and with well-being (secondary outcomes).

In an additional set of analyses, we computed the prevalence of stigma using alternate definitions (see Stigma definition) and assessed associations of these with poor glycemic control. We also evaluated associations of our main stigma definition with individual components of the poor glycemic control definition.

## Results

### Participants

Recruitment targets were achieved (384 respondents; 4 removed because of implausible answers or time to completion) with representation from all 10 Canadian provinces. Participants were largely of European origin (351/380, 92.4%) and English-speaking (315/380, 82.9%; see Table 1). Average age was 19.5 years (SD 3.3), and 46.8% (178/380) were aged between 14 and 18 years. In terms of gender identity, 257 were girls or women (67.6%), 118 were boys or men (31.1%), and 5 indicated being agender or gender fluid (1.3%). With respect to sexual orientation, the majority were heterosexual (302/380, 79.5%), 8.9% (34/380) were bisexual, 5.3% (20/380) were homosexual, 3.4% (13/380) did not know yet, and 2.6% (10/380) preferred not to respond.

**Prevalence**

The prevalence of stigma (Table 2) was 65.5% (95% CI 60.7%-70.3%) by our primary definition (ie, at least one of the 3 most stigma-relevant BDA questions). About two-thirds described a personal experience of stigma (63.4%; 95% CI 58.6%-68.3%). The prevalence of stigma by our primary definition was slightly higher among girls at 68.3% (95% CI 62.7%-74.0%) compared with 59.3% (95% CI 50.3%-68.3%) among boys. Similarly, the proportion reporting stigma by our primary definition was slightly higher among young adults (aged 19-24 years) at 69.3% (95% CI 62.9%-75.7%) than among adolescents at 61.2% (95% CI 54.1%-68.5%).

### Glycemic Control

An HbA_1c_ value was available for 312 out of 380 participants (82.1%). This included 112 with both mailed-in capillary blood samples for direct measurement and self-reported recent HbA_1c_, 26 with the mailed-in sample only, and 174 with the self-reported HbA_1c_ only. Among those with both direct and reported HbA_1c_ measures (n=112), moderate agreement was observed (*r*=.41, 95% CI 0.25-0.56; Bland-Altman plot; see Multimedia Appendix 1). By our primary definition, poor glycemic control was observed in 36.9% (95% CI 31.5%-42.2%) of the 312 participants for whom HbA_1c_ was available; among these individuals, 17 out of 312 (5.4%) participants had both an HbA_1c_>9% and at least one severe hypoglycemia in the past year, 64 out of 312 had experienced severe hypoglycemia without HbA_1c_>9% (20.5%, 95% CI 16.0%-25.0%), and 34 out of 312 had an HbA_1c_>9% (10.9%, 95% CI 7.4%-14.4%) without severe hypoglycemia.

**Table 1:** Respondent characteristics.

Characteristics		All	Adolescents^a^	Young adults^b^
Number of respondents		380	178	202
**Gender, n (%)**				
	Male	118 (31.1)	74 (41.6)	44 (21.8)
	Female	257 (67.6)	101 (56.7)	156 (77.2)
	Other	5 (1.3)	3 (1.7)	2 (1.0)
Age, years, mean (SD)		19.5 (3.3)	16.3 (1.3)	22.2 (1.7)
Age at diagnosis, in years, mean (SD)		9.9 (5.3)	8.3 (4.2)	11.3 (5.8)
Diabetes duration, in years, mean (SD)		9.6 (5.4)	8.1 (4.5)	10.9 (5.8)
Currently in school, n (%)		271 (71.5)	155 (87.1)	116 (57.4)
Living with parents, n (%)		264 (69.5)	167 (93.8)	97 (48.0)
Insulin pump, n (%)		220 (57.9)	102 (57.3)	118 (58.4)
HbA_1c_^c^, mean (SD)		7.8 (1.7)	7.9 (1.4)	7.8 (1.9)
Self-reported hypoglycemic episodes per week, mean (SD)		3.2 (2.3)	3.5 (2.5)	3.0 (2.0)
Having experienced severe hypoglycemia in the last year, n (%)		106 (27.9)	48 (27.0)	58 (28.7)

^a^14-18 years.

^b^19-24 years.

^c^HbA_1c_ was available for 312 participants.

**Table 2:** Prevalence of stigma with 95% CIs. BDA: Barriers to Diabetes Adherence in Adolescence questionnaire

Definition		Prevalence (95% CI)				
		All	Girls^a^	Boys^a^	Adolescents	Young adults
**Primary definition**						
	Endorsed ≥1 of the 3 most relevant items^b^ of the BDA stigma subscale	65.5% (60.7-70.3)	68.6% (62.9-74.3)	59.3% (50.3-68.3)	61.2% (54.0-68.5)	69.3% (62.9-75.7)
**Other definitions**						
	Provided an example	63.4% (58.6-68.3)	70.5% (64.9-76.1)	47.5% (38.3-56.6)	51.7% (44.3-59.1)	73.8% (67.6-79.9)
	Endorsed ≥1 of the 3 most relevant items^b^ of the BDA stigma subscale and provided an example	47.1% (42.1-52.2)	51.2% (45.1-57.2)	38.1% (29.2-47.0)	39.3% (32.1-46.6)	54.0% (47.0-60.9)

^a^According to their sex at birth.

^b^I try not to deal with my diabetes in front of friends. I have a hard time telling people I have diabetes. I feel embarrassed taking care of my diabetes in front of other people.

### Associations of Stigma With Poor Glycemic Control

Among participants fulfilling our primary definition of stigma, the odds of poor glycemic control were more than twice as high as in the remaining respondents in both unadjusted (odds ratio [OR] 2.32, 95% CI 1.39-3.89) and adjusted models (OR 2.25, 95% CI 1.33-3.80; adjusted for age, sex, and type of treatment). The odds of HbA_1c_>9% (>75 mmol/mol) was 3-fold greater (OR 3.05, 95% CI 1.36-6.86), and the odds of severe hypoglycemic episode in the past year was nearly 2-fold greater (OR 1.86, 95% CI 1.05-3.31) in those with versus without stigma (adjusted models; Table 3). Alternate stigma definitions demonstrated an approximately 2-fold higher odds of poor glycemic control.

In the alternate model including self-efficacy and well-being as independent variables, OR for poor glycemic control with versus without stigma was 1.82 (95% CI 1.06-3.13). A 1-point increase in the self-efficacy score (10-point scale) was associated with a 20% lower risk for poor glycemic control (OR 0.8, 95% CI 0.6-0.9); on average, the scores were 6.5 (SD 1.7) with stigma and 7.6 (SD 1.6) without using the primary stigma definition (see Multimedia Appendix 1). There was no association between well-being and poor glycemic control (OR 1.0, 95% CI 0.98-1.01).

### Associations of Stigma (by the Primary Definition) With Perceived Well-Being and Self-Efficacy for Diabetes Self-Management

In an adjusted linear regression model (age, sex, type of treatment; Table 4), having stigma (by the primary definition) was associated with a 7.5-point lower score (95% CI −11.8 to −3.3) on the Well-Being index (range 0-100). The threshold for a clinically relevant change is considered to be 10 points [24].

In a separate model, having stigma was associated with a 0.9 (95% CI −1.3 to −0.6) lower SEDM scale score (range 1-10), which corresponds to an approximately 0.5 SD lower score (Table 5).

**Table 3:** Association between stigma and glycemic control, odds ratios with 95% CI. BDA: Barriers to Diabetes Adherence in Adolescence questionnaire; OR: odds ratio.

Stigma definition	Glycated hemoglobin, HbA_1c_>9% (75 mmol/mol), OR (95% CI)		Self-reported ≥1 severe hypoglycemia in the previous year, OR (95% CI)		Poor glycemic control overall, OR (95% CI)	
	Univariate	Multivariate^a^	Univariate	Multivariate^a^	Univariate	Multivariate^a^
A: BDA 3 most relevant (at least one of the 3 items)	3.39 (1.53-7.51)	3.05 (1.36-6.86)	1.76 (1.00-3.09)	1.86 (1.05-3.31)	2.32 (1.39-3.89)	2.25 (1.33-3.80)
Item 1: I try not to deal with my diabetes in front of friends	2.58 (1.39-4.77)	2.62 (1.39-4.94)	1.67 (1.00-2.77)	1.74 (1.04-2.91)	2.02 (1.27-3.23)	2.03 (1.27-3.27)
Item 2: I have a hard time telling people that I have diabetes	1.32 (0.72-2.41)	1.11 (0.59-2.07)	1.64 (0.99-2.73)	1.77 (1.05-3.00)	1.56 (0.98-2.48)	1.48 (0.92-2.38)
Item 3: I feel embarrassed taking care of my diabetes in front of other people	2.34 (1.24-4.44)	2.03 (1.04-3.93)	1.52 (0.91-2.54)	1.63 (0.96-2.76)	1.62 (1.02-2.58)	1.54 (0.96-2.49)
B: Personal experience (open-ended question)	2.47 (1.18-5.14)	2.18 (1.03-4.65)	1.26 (0.73-2.16)	1.38 (0.78-2.43)	1.62 (0.99-2.68)	1.58 (0.94-2.65)
A and B: (BDA stigma 3 most relevant + personal experience)	2.69 (1.42-5.11)	2.44 (1.27-4.72)	1.66 (0.99-2.77)	1.79 (1.06-3.03)	2.06 (1.29-3.29)	2.02 (1.25-3.26)

^a^Multivariate: age, sex at birth, type of treatment (multiple daily injection or insulin pump).

**Table 4:** Linear regression model evaluating association between stigma and well-being, adjusted for age, sex, and use of insulin pump.

Variables	Change in well-being score (95% CI)
Stigma presence (primary definition)	−7.5 (−11.80 to -3.26)
Treatment (insulin pump)	3.7 (−0.39 to 7.80)
Age	−0.28 (−0.90 to 0.35)
Sex (female)	−3.2 (−7.68 to 1.28)

**Table 5:** Linear regression model evaluating association between stigma and self-efficacy, adjusted for age, sex, and use of insulin pump.

Variables	Change in self-efficacy score (95% CI for B)
Stigma presence (primary definition)	−0.90 (−1.25 to −0.55)
Treatment (insulin pump)	0.42 (0.09 to 0.76)
Age	−0.08 (−0.13 to −0.03)
Sex (female)	−0.61 (−0.97 to −0.24)

## Discussion

### Principal Findings

Among 380 youth with type 1 diabetes recruited through social media from across Canada, the prevalence of some degree of stigma was approximately 65% (ie, endorsement of at least one of 3 key items on the BDA stigma subscale). Youth with some degree of stigma were more likely to have poor glycemic control. They were twice as likely to have either an HbA_1c_ above 9% or one or more hypoglycemic events in the prior year. When these components of poor glycemic control were considered separately, youth with stigma were 3 times as likely to have an HbA_1c_ above 9%, and they were twice as likely to have had a hypoglycemic event in the prior year. Stigma was also associated with a lower sense of well-being and less self-efficacy for diabetes management. Our findings are a call to action to develop, test, and implement strategies to address stigma in youth with type 1 diabetes.

Previous qualitative studies of stigma in type 1 diabetes [3,8,25,26] provide important insights into the roots and experiences of stigma, but cannot capture the prevalence of the problem, in contrast to our study. To recruit the participants, we opted to partner with diabetes-related organizations to reach them through social media. In 2015, 100% of Canadian young adults had internet access [27]. Over 80% of Canadian youth report daily use of social networking sites [28]. The use of social media combined with an online questionnaire allowed us to meet recruitment targets at relatively low cost and to attract respondents throughout Canada, a country with an area of 10 million km². Our success with this approach is consistent with the findings from a systematic review evaluating social media–based recruitment of youth into health research studies [29]. However, there is a possibility that such a recruitment approach may also attract fictitious respondents or responses. To mitigate this possibility, study promotion occurred only through diabetes-specific organizations and incentives were not publicized. Furthermore, we examined all responses in terms of time to completion and variability in responses; the responses of 4 individuals appeared suspect in this regard and were excluded. We offered a response incentive to encourage survey completion, apparent when the survey was started. A meta-analysis reported that participants who start a survey are more likely to finish it (OR 1.27, 95% CI 1.12-1.44) if an incentive is offered [30].

The BDA questionnaire was specifically designed for adolescents and addresses consequences of stigma through one of its subscales [20]. For our main stigma definition, we made an a priori decision to use endorsement of one or more of the 3 BDA items that appeared to most directly query stigma. We also tested several alternative definitions, and the prevalence of stigma was consistently in the order of 60%. Almost half of respondents (47%) were captured by all definitions. We opted not to include the other 3 items of the BDA stigma subscale as we considered these to be reflective more of personal difficulties or challenges with diabetes management rather than necessarily being stigma-related. For example, “Restaurants are challenging for me” could be a consequence of not knowing the carbohydrate content of the different dishes offered. Other items were “Parties and social gatherings get in the way of taking care of my diabetes and I need to find a private place to take care of my diabetes.”

The two-thirds of participants whom we estimate to have some degree of stigma is similar to the proportion reported in a Swiss study, largely among adults (median age 67 years, ranging from 16 to 96 years) with type 1 or type 2 diabetes. Their definition was having been discriminated against because of their health condition [17]. Their estimate rose to 85% when stigma was defined as perceiving at least one stereotypical attribution [17]. In an adult population with type 1 diabetes, 74% of the respondents believed that diabetes comes with social stigma [18]. We observed a slightly higher prevalence of stigma, according to the primary definition, among young adults than among adolescents (69% vs 61%). The transition from pediatric to adult care occurs concurrently with the increase in stigma prevalence. Addressing stigma before or during this transition may be a means of countering the deterioration in glycemic control that typically occurs at this time.

Stigma was observed in a higher proportion of girls than boys (69% vs 59% by the primary definition). It has been reported that girls with type 1 diabetes feel more embarrassed by their disease [26] and have more diabetes-related distress [31] and higher rates of diabetes-related acute complications [32] and hospitalizations [33]. In contrast to prior studies, we queried not only biological sex but also gender identity; however, the low number of participants who considered themselves to be neither a boy nor a girl did not allow specifically estimation of stigma prevalence in this subgroup of individuals. In terms of sexual orientation, 20% of participants reported being homosexual, bisexual, or did not know yet; there were too few individuals in each category to draw conclusions on the impact of sexual orientation on stigma prevalence or associations with glycemic control. Examination of stigma prevalence and impact in these subpopulations of individuals with type 1 diabetes deserves dedicated study given that “being different” in terms of gender identity or sexual orientation may compound the experience of diabetes-related stigma [34]. In terms of ethnocultural representation, the sample was preponderantly of European origin but that is consistent with the epidemiology of type 1 diabetes [35], in contrast to type 2 diabetes. Studies on prevalence of stigma have addressed this issue mainly in (>90%) individuals of European origin [17,18]. Specific studies in individuals from other ethnic backgrounds are needed.

We evaluated the associations between stigma and glycemic control in youth. Importantly, our definition for poor control combined a high HbA_1c_ with severe hypoglycemia, defined as having at least one episode requiring assistance from someone else in the past year. Higher HbA1c levels are associated with greater risk for diabetes-related complications, with the merits of “tight” control demonstrated in the Diabetes Control and Complications Trial [36]. However, lower HbA_1c_ levels cannot be traded off for increased risk of severe hypoglycemia, with its attendant risk of death, loss of consciousness, and other injury [37,38]. Navigating the space between hypoglycemia and hyperglycemia is particularly difficult in type 1 diabetes, in contrast to type 2 diabetes. We determined that stigma was associated with a 2.3-fold higher odds of poor glycemic control, defined as having an HbA_1c_ over 9% (75 mmol/mol) and/or having experienced a severe hypoglycemic episode in the previous year. Importantly, stigma was conclusively associated not only with this composite measure of poor control but also with its individual components. These associations were robust across various definitions of stigma evaluated (Table 3). Our study is the first to demonstrate a clear association between stigma and both hyperglycemia and hypoglycemia in youth with type 1 diabetes.

We demonstrated a negative association with SEDM, as captured by the SEDM measure. This is consistent with qualitative studies that have reported that patients neglect diabetes care to avoid stigma. For example, in public places, people will skip blood glucose testing or will delay insulin injections [12,39]. In our sample, stigma was associated with lower scores for every item on the self-efficacy scale used. Our survey did not directly capture self-care behavior, but lower self-efficacy has been shown to be associated with poorer diabetes self-management [40]. Indeed, in the logistic regression model evaluating SEDM alongside stigma in terms of associations with glycemic control, lower self-efficacy was associated with poor glycemic control.

It is important to emphasize that the importance of addressing stigma lies not only in optimizing glycemic control but also in enhancing overall well-being. Indeed, in people with diabetes, emotional well-being may be compromised by the burden of living with diabetes [41]. In this study, respondents with stigma reported a lower sense of well-being. In the Swiss study of adults with type 1 or type 2 diabetes previously discussed, respondents with stigma also reported higher levels of psychological distress [17]. Our identification of high stigma prevalence and a low sense of overall well-being in youth with type 1 diabetes is clearly important; addressing stigma may be one avenue to emotionally support youth with type 1 diabetes.

### Limitations

Our study has some limitations. Given its cross-sectional nature, causality cannot be proven. Indeed, it is likely that there is a bidirectional effect of stigma and glycemic control such that stigma adversely affects glycemic control, and poor control contributes to stigma (eg, hypoglycemic episodes witnessed by others, high HbA_1c_ levels known to health care providers and family). We were able to estimate the prevalence of stigma in a large sample, but we cannot be certain that this sample is representative of all youth with type 1 diabetes. Those with stigma may have been more likely to participate. In comparison to prior studies, we queried gender identity and sexual orientation, but these subgroups of individuals were too small to study specifically. Not all participants provided a mailed-in capillary blood sample; however, there was a reasonable correlation between measured and reported HbA_1c_ in those who provided both. Some respondents may have provided fictitious answers; however, we endeavored to mitigate this through various approaches previously described.

### Conclusions

Despite some limitations, our study provides important findings. Stigma is prevalent in youth with type 1 diabetes and is associated with lower diabetes-related self-management self-efficacy, high HbA_1c_ levels, severe hypoglycemia, and diminished sense of well-being. Our findings indicate that stigma can be captured through a few simple questions. These results should stimulate clinicians, friends, and family members to ask about stigma and work toward addressing it to help youth with type 1 diabetes avoid diabetes-related complications and lead happier and safer lives.

## Appendix

Multimedia Appendix 1 STIGMA supplementary material.

## Abbreviations

BDA: Barriers to Diabetes Adherence in Adolescence questionnaire
HbA_1c_: glycated hemoglobin
OR: odds ratio
SEDM: self-efficacy for diabetes self-management
