# Supplementary material for: Stigma and Its Association With Glycemic Control and Hypoglycemia in Adolescents and Young Adults With Type 1 Diabetes: Cross-Sectional Study
Source: J Med Internet Res. 2018 Apr 20;20(4):e151. doi: 10.2196/jmir.9432 (PMC5935805; doi:10.2196/jmir.9432)
Supplement: Multimedia Appendix 1 [file jmir_v20i4e151_app1.pdf]

Table 1 Diabetes organisations and groups used for recruitment

**Support groups and organization**	**About the organization**
Alberta Children's Hospital & transition clinics	Children's hospital in Calgary
British Columbia Children's hospital	Children's hospital in Vancouver
Calgary Insulin Pumpers (Alberta)	Support group for those interested in T1D education
Canadian Celiac Association	Dedicated to improving diagnosis and quality of life for those adversely affected by gluten
Canadian Obesity Network	Largest obesity association in Canada
Celiac association NL & Labrador chapter	Newfound Land chapter of the Canadian Celiac Association
Celiac Association of Canada Calgary	Calgary chapter of the Canadian Celiac Association (Alberta)
Connected In Motion	T1D support group (Ontario)
Crohn's and Colitis Canada	National, volunteer-based charity focused on finding the cures for Crohn’s disease and ulcerative colitis
Diabète Québec	Quebec association that wants to educate people with diabetes
Diabetes Canada	National voice for those living with diabetes or prediabetes
Dskate Canada	Hockey and diabetes camp (Quebec and Ontario)
Hôpital Ste-Justine	Children's hospital in Montreal, Quebec
Live Well! Bien Vivre!	Prevention & management of chronic medical conditions with the help of wellness coaches;
National Aboriginal Diabetes Association	Trying to prevent and manage diabetes by working with people, communities and organizations
Southern Ontario Aboriginal Diabetes Initiative	supporting Indigenous communities, families and individuals to make healthy choices and live free of diabetes
The Canadian Addison Society	Organization that wants to educate people about Addinson’s disease (Ontario)
Vancouver Island Addison support group	Vancouver chapter of the Cnadian Addison Society, British Columbia
Waltzing the dragon	Website’s aim is to educate, equip and encourage families dealing with type 1 diabetes (Alberta)
Waterloo Wellington Diabetes	Central intake for referrals to diabetes education & specialists, Ontario
Young & T1	T1D support group, British Columbia
Athletes+causes	Helps athletes do their philanthropic work to educate and instill hope in others
Beyondtype1.org	Supportive community via social media
ConsultantPtCare	An online community bridging the gap between health care and patient care
Diabetes Care Community	Network of people with diabetes and their support network involved in diabetes care
Diabetic connect	World's largest online social network for people & families living w/ diabetes
T1dactiveliving	Her honest personal journey with T1D in hopes of inspiring others to be strong
TheCeliacScene	Your premier source for gluten-free living
Type 1 Diabetes Think Tank Network	This online group tries to improve the quality of life & health outcomes for people with T1D
type1diabetesproject	Helps people with T1D live healthier lives
Type1strong	Raising awareness that you can be strong & healthy even though have T1D
Uwalk	An interactive website that motivates ppl to exercise
Abbott Freestyle	Online community committed to helping people with diabetes - run by Abbott Laboratories
Accu-Chek Canada	A company that makes glucose meters
Banting House	Creates public awareness of the national historic significance of Sir Frederick Banting
Canadian Diabetes Care Guide	Digest-sized Canadian magazine written by health care professionals
Centre for Studies in Primary Care (CSPC)	The research arm of the Department of Family Medicine at Queen's University, Ontario

Champlain Diabetes Services	Resource for people living with diabetes & for healthcare providers in the Champlain region, Ontario
Diabetes Care Program of Nova Scotia	Using leadership to improve the health of those living with or at risk of developing diabetes
Dietitians of Canada	Professional association representing 6,000 members across Canada
Guelph General Hospital	A hospital found in Guelph, Ontario
Health Prince Edward Island	Delivers publicly funded health services
Horizon Health network for the Maritimes	A health network of hospitals and clinics in the Maritimes (New Brunswick)
Hotel Dieu Hospital	Hospital in Kingston, Ontario
Huron Perth Diabetes Education Program	Diabetes program offered at 7 different hospitals in the Huron-Perth, Ontario
IWK Health Centre	Pediatric and obstetric care in the Maritime provinces (Nova Scotia)
Joseph Brant Hospital	A hospital found in Burlington, Ontario
Live Well! Bien Vivre!	Wellness programs and experienced health coaches available to help pts achieve a healthier lifestyle that is funded by Diabetes Canada (New Brunswick)
Markham Stouffville Hosp	A hospital found in Markham, Ontario
Medavie Blue Cross	Not for profit Canadian medical insurance company
Medtronics Canada	A company that makes insulin pumps
MYSUGR	An application for diabetes management (USA)
Niagara Health System	Found in the Niagara region, it is Ontario's largest multi-site hospital amalgamation
Norfolk General Hospital	A general hospital in Simcoe, Ontario
Provincial Council for Maternal and child health	Provincial (Ontario) forum where leaders in the field can identify issues of importance in health care
Rouge Valley Health System	A hospital corporation based in the Greater Toronto area, Ontario
St. Joe's Youth Wellness Centre	An accessible centre in Hamilton, (Ontario) for people aged 17 to 25 to receive care for mental health and addiction issues
Stollery Children's Hospital	A children's hospital in Edmonton, Alberta
Trillium Health Partners	A hospital system which serves Mississauga and western Toronto, Ontario

Table 2 Prevalence of stigma according to different cut-points on the BDA stigma-subscale and associations with glycemic control

	Prevalence	Association with glycemic control ‡		
**Cut-off on the 5 point likert-type BDA-*stigma subscale* ***	**Endorsing one of the 3 most relevant items†**<br>%<br>[95% CI]	**A1C >9%**<br>OR<br>[95% CI]	**Self-reported ≥1 severe hypoglycemia in the previous year**<br>OR<br>[95% CI]	**Poor glycemic control overall**<br>OR<br>[95% CI]
Score ≥ 2	65.5%<br>[60.7-70.3]	3.05<br>[1.36-6.86]	1.86<br>[1.05-3.31]	2.25<br>[1.33-3.80]
Score ≥ 3	44.0%<br>[38.9-49.0]	2.16<br>[1.14-4.08]	1.83<br>[1.08-3.11]	1.97<br>[1.22-3.19]
Score ≥ 4	28.7%<br>[24.1-33.3]	1.99<br>[1.05-3.78]	2.44<br>[1.40-4.24]	2.13<br>[1.27-3.56]
Score = 5	14.0%<br>[10.5-17.5]	2.01<br>[0.92-4.42]	2.24<br>[1.10-4.57]	2.64<br>[1.33-5.26]

* The 5-point likert-type scale ranged from 1- not at all true to 5- completely true

† The 3 most relevant items: I try not to deal with my diabetes in front of friends. I have a hard time telling people I have diabetes. I feel embarrassed taking care of my diabetes in front of other people.

‡ Multivariate logistic regression models adjusted for age, sex at birth, type of treatment (multiple daily injection or insulin pump)

Table 3 Self-efficacy for diabetes management

**Self-efficacy for diabetes self-management (SEDM) Items**	**Being affected by stigma n=249**	**Not affected by stigma n=131**	
	Mean score (sd)	Mean score (sd)	Mean difference [95% CI]
Adjust insulin when you eat more or less than usual	7.8 (2.0)	8.4 (2.1)	0.6 [0.2-1.0]
Choose healthy foods when you go out to eat	7.2 (2.3)	7.8 (2.4)	0.6 [0.1-1.1]
Exercise even when you don’t really feel like it	5.8 (2.9)	6.9 (3.0)	1.1 [0.5-1.7]
Adjust insulin or food accurately based on how much exercise you get	6.4 (2.6)	7.4 (2.6)	1.0 [0.5-1.6]
Talk to your doctor or nurse about any problems you are having with taking care of my diabetes	7.2 (2.7)	8.4 (2.3)	1.3 [0.7-1.8]
Do your blood sugar checks even when you are really busy	6.1 (2.8)	7.4 (2.6)	1.2 [0.7-1.8]
Manage your diabetes the way your health care team wants you to	6.1 (2.6)	7.1 (2.7)	0.9 [0.4-1.5]
Manage your diabetes even when you feel overwhelmed	6.0 (2.7)	7.3 (2.6)	1.4 [0.8-1.9]
Find ways to deal with feeling frustrated about your diabetes	5.6 (2.7)	7.1 (2.7)	1.5 [0.9-2.02]
Identify things that could get in the way of managing your diabetes	7.2 (2.1)	8.2 (2.1)	0.9 [0.5-1.4]
SEDM Total score	6.5 (1.7)	7.6 (1.6)	1.1 [0.7-1.4]

Maximal score is 10 indicative of greater self-efficacy

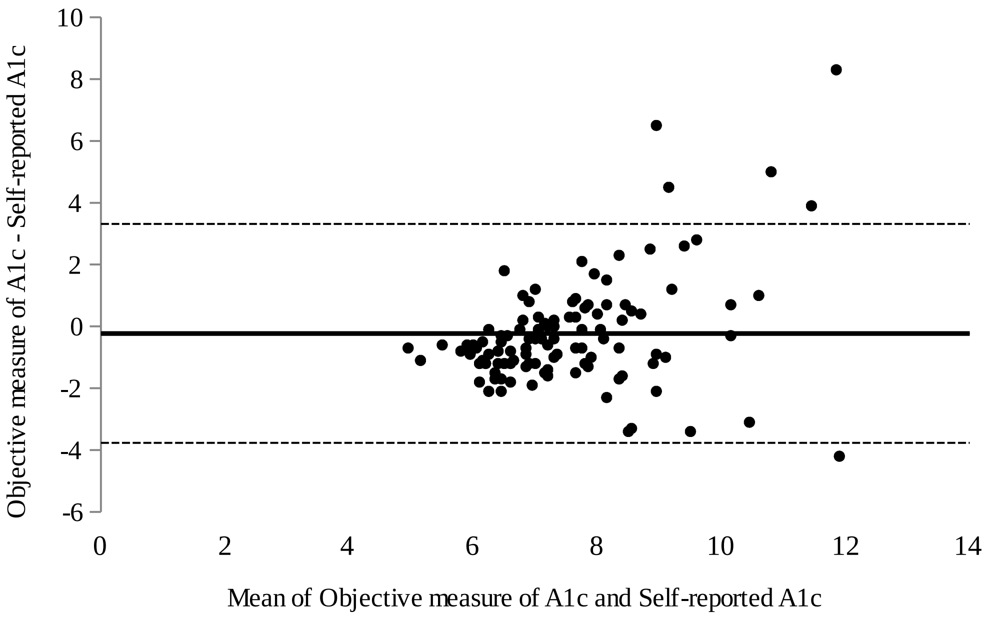

Figure 1. Bland-Altman plot for agreement between objectively measured and self-reported A1c
